# Geriatric Assessment in CKD Care: An Implementation Study

**DOI:** 10.1016/j.xkme.2024.100809

**Published:** 2024-03-21

**Authors:** Carlijn G.N. Voorend, Noeleen C. Berkhout-Byrne, Leti van Bodegom-Vos, Adry Diepenbroek, Casper F.M. Franssen, Hanneke Joosten, Simon P. Mooijaart, Willem Jan W. Bos, Marjolijn van Buren, POLDER study group, Arjan van Alphen, Noeleen Berkhout-Byrne, Fenna van Breda, Marjolijn van Buren, Henk Boom, Willem Jan Bos, Adry Diepenbroek, Marielle Emmelot-Vonk, Casper Franssen, Carlo Gaillard, Nel Groeneweg-Peeters, Bettie Hoekstra, Nienke Hommes, Francoise Hoornaar, Hanneke Joosten, Joep Lagro, Elisabeth Litjens, Femke Molenaar, Simon Mooijaart, Aegida Neradova, Mike Peters, Michelle Troost, Wilma Veldman, Carlijn Voorend, Lidwien Westerbos, Carlijne Westerman-van der Wijden, Judith Wierdsma

**Affiliations:** 9 Maasstad Hospital, Rotterdam; 10 Leiden University Medical Center, Leiden; 11 Amsterdam University Medical Center, Amsterdam; 12 Haga Hospital, The Hague; 13 Reinier de Graaf Hospital, Delft; 14 St. Antonius Hospital, Nieuwegein; 15 University Medical Center Groningen, Groningen; 16 University Medical Center Utrecht, Utrecht; 17 University Medical Center Groningen, Groningen; 18 University Medical Center Utrecht, Utrecht; 19 Reinier de Graaf Hospital, Delft; 20 Maasstad Hospital, Rotterdam; 21 Haaglanden Medical Center, The Hague; 22 St. Antonius Hospital, Nieuwegein; 23 Maastricht University Medical Center +, Maastricht; 24 Haga Hospital, The Hague; 25 Maastricht University Medical Center +, Maastricht; 26 University Medical Center Utrecht, Utrecht; 27 Leiden University Medical Center; 28 Amsterdam University Medical Center, Dianet, Amsterdam; 29 University Medical Center Utrecht, Utrecht; 30 Reinier de Graaf Hospital, Delft; 31 University Medical Center Groningen, Groningen; 32 Leiden University Medical Center; 33 Amsterdam University Medical Center, Amsterdam; 34 Haaglanden Medical Center, The Hague; 35 University Medical Center Utrecht, Utrecht; 1 Department of Internal Medicine (Nephrology), Leiden University Medical Center, Leiden, The Netherlands; 2 Department of Biomedical Data Sciences, Section Medical Decision Making, Leiden University Medical Center, Leiden, The Netherlands; 3 Department of Nephrology, University Medical Centre Groningen, University of Groningen, Groningen, The Netherlands; 4 Department of Internal Medicine, Division of General Internal Medicine, Section Geriatric Medicine, Maastricht University Medical Center+, Maastricht, The Netherlands; 5 Department of Gerontology and Geriatrics, Leiden University Medical Center, Leiden, The Netherlands; 6 LUMC Center for Medicine for Older People, Leiden University Medical Center, Leiden, The Netherlands; 7 Department of Internal Medicine St. Antonius Hospital, Nieuwegein, The Netherlands; 8 Department of Internal Medicine, Haga Hospital, The Hague, The Netherlands

**Keywords:** Chronic kidney disease, feasibility studies, geriatric assessment, implementation science, older people, shared decision making

## Abstract

**Rationale & Objective:**

Older people with progressive chronic kidney disease (CKD) have complex health care needs. Geriatric evaluation preceding decision making for kidney replacement is recommended in guidelines, but implementation is lacking in routine care. We aimed to evaluate implementation of geriatric assessment in CKD care.

**Study Design:**

Mixed methods implementation study.

**Setting & Participants:**

Dutch nephrology centers were approached for implementation of geriatric assessment in patients aged ≥70 years and with an estimated glomerular filtration rate of ≤20 mL/min/1.73 m^2^.

**Quality Improvement Activities/Exposure:**

We implemented a consensus-based nephrology-tailored geriatric assessment: a patient questionnaire and professionally administered test set comprising 16 instruments covering functional, cognitive, psychosocial, and somatic domains and patient-reported outcome measures.

**Outcomes:**

We aimed for implementation in 10 centers and 200 patients. Implementation was evaluated by (i) perceived enablers and barriers of implementation, including integration in work routines (Normalization Measure Development Tool) and (ii) relevance of the instruments to routine care for the target population.

**Analytical Approach:**

Variations in implementation practices were described based on field notes. The postimplementation survey among health care professionals was analyzed descriptively, using an explanatory qualitative approach for open-ended questions.

**Results:**

Geriatric assessment was implemented in 10 centers among 191 patients. Survey respondents (n = 71, 88% response rate) identified determinants that facilitated implementation, ie, multidisciplinary collaboration (with geriatricians) -meetings and reports and execution of assessments by nurses. Barriers to implementation were patient illiteracy or language barrier, time constraints, and patient burden. Professionals considered geriatric assessment sufficiently integrated into work routines (mean, 6.7/10 ± 2.0 [SD]) but also subject to improvement. Likewise, the relevance of geriatric assessment for routine care was scored as 7.8/10 ± 1.2. The Clinical Frailty Score and Montreal Cognitive Assessment were perceived as the most relevant instruments.

**Limitations:**

Selection bias of interventions’ early adopters may limit generalizability.

**Conclusions:**

Geriatric assessment could successfully be integrated in CKD care and was perceived relevant to health care professionals.

## Plain-Language Summary

The number of older persons with kidney failure is increasing, many of whom have cognitive decline or are dependent on others for daily life tasks. These problems are often overlooked but relevant for future treatment choices, and they affect quality of life. We asked 10 health care centers to use tests and questionnaires to identify these issues, thus being able to offer additional support. We learned that it is possible to use these assessments in practice and that professionals found them relevant. Collaboration with geriatric departments was perceived valuable. However, there are also challenges, such as not having enough time and personnel and burden to patients. Understanding these possibilities and challenges is crucial for improving care for older patients with kidney failure.

Among the increasing population of older patients with kidney failure,^1^^,^^2^ unrecognized geriatric impairments are highly prevalent. Such impairments, including cognitive and functional decline, comorbid conditions, frailty, depression, and malnutrition,^3^, ^4^, ^5^ are associated with adverse health outcomes such as mortality, hospitalization, and reduced health-related quality of life.^6^^,^^7^ Understanding geriatric impairments can be valuable for decision making for kidney replacement therapy choices and for risk stratification. Therefore, Dutch and British guidelines recommend geriatric assessment for those at high risk of death or those identified as being frail, respectively.^8^^,^^9^ For this, clinicians have recognized the clinical and scientific value of a standardized set of instruments.^7^^,^^10^

In the absence of a gold standard for geriatric assessment, a group of Dutch health care professionals recently reached a consensus on a geriatric assessment tailored for nephrology care (nephrology-tailored geriatric assessment [NGA]).^11^ This set of instruments takes less than 1 hour to perform and could be routinely conducted by trained nephrology or geriatric nurses. In contrast to comprehensive geriatric assessment, this modified approach of geriatric assessment is therefore expected to be less challenging to embed in existing routines in nephrology care pathways. Furthermore, unlike short frailty screening instruments,^12^ the set would enable adequate recognition of geriatric impairments or frailty in the older chronic kidney disease (CKD) population. After NGA, if needed, a patient could be referred to a geriatrician for full comprehensive geriatric assessment, which includes a more extensive multidisciplinary diagnostic and treatment process that identifies medical, psychosocial, and functional impairments that may necessitate development of an integrated care plan.

Although some previous attempts at incorporating a geriatric assessment in nephrology populations have been reported,^3^^,^^4^^,^^13^, ^14^, ^15^, ^16^ consistent and widespread implementation of standardized geriatric assessment in routine nephrology care has yet to be achieved. Analysis of the process of implementation (including barriers and facilitators) may help to successfully incorporate this complex intervention in clinical practice.^17^ The current study, therefore, aimed to evaluate multicenter implementation of geriatric assessment in routine nephrology care.

## Methods

### Design

In this implementation study, we used a mixed methods approach, combining quantitative data collection with a partially qualitative postimplementation survey involving health care professionals.

### Context

This study was part of the Pathway for Older Patients Reaching End-Stage Renal Disease (POLDER) initiative, aimed at designing, implementing, and evaluating geriatric assessment in Dutch nephrology clinics. In October 2017, we approached 16 Dutch university and non–university hospital–based nephrology centers on their interest in implementing geriatric assessment. This interest was evidenced either by partaking in previous studies involving geriatric assessment or by showing interest in the topic at conferences. Following an email survey to assess current practices,^11^ 10 centers chose to participate, representing 18% of all Dutch nephrology centers. Although the geriatric assessment protocol was new for all participating centers, some had previously participated in studies using geriatric assessment in patients with CKD stage G4 or G5 (N = 3) or CKD G5 or G5D (N = 2).^3^^,^^18^ Additionally, 2 centers used geriatric assessment in their routine care on referral, whereas the remaining 3 centers had not previously used geriatric assessment in CKD care.

### Intervention Description

Previously, we developed a consensus-based nephrology-tailored assessment (NGA).^11^ NGA consists of a patient questionnaire and professionally administered tests that can be completed within 1 hour by a trained nurse or medical specialist. NGA includes instruments covering various domains, including functional, cognitive, psychosocial, somatic, and patient-reported outcomes. Table 1 presents the included instruments^19^, ^20^, ^21^, ^22^, ^23^, ^24^, ^25^, ^26^, ^27^, ^28^, ^29^, ^30^, ^31^, ^32^, ^33^ and by whom they were completed. NGA was developed to enhance routine care and research in older patients with advanced CKD, aiming to identify known and unknown geriatric impairments in older patients with CKD stage G4-G5. If needed, NGA could lead to appropriate supportive interventions (eg, physiotherapy, referral to geriatrician) and is beneficial to decision making in patients with kidney failure.

**Table 1:** Instruments Included in the Nephrology-tailored Geriatric Assessment

Nephrology-Tailored Geriatric Assessment: Domains and Instruments				Type of Assessment	
Domain		Subdomain	Instrument	(I) Patient questionnaire	(II) Provider-administered test set
	Functional status	Activities of daily living	Katz Activities of Daily Living-6^19^	X	
		Instrumental activities of daily living	Lawton Instrumental Activities of Daily Living^20^	X	
		Handgrip strength	Handgrip strength		X
		Fall risk assessment	One-year fall history, fear of falling		X
	Cognitive status	Cognitive functioning	Montreal Cognitive Assessment^21^		X
			6-item Cognitive Impairment Test^22^		X
			Letter Digit Substitution Test^23^		X
	Psychological status/mood	Depression	Whooley-questions/Geriatric Depression Scale 15-item^24^^,^^25^		X
		Optimism	Life Orientation Test-Revised^26^	X	
	Patient-reported outcome measures	Health-related quality of life	12-item Short Form Health Survey^27^	X	
		Symptoms	Dialysis Symptom Index^28^	X	
	Somatic status	Clinical judgment	Surprise question^29^		X
		Frailty	Clinical Frailty Score^30^		X
		Comorbid condition	Charlson Comorbidity Index^31^		X
		Polypharmacy	Polypharmacy (≥5 medications)		X
		Nutritional status	Patient-Generated Subjective Global Assessment^32^	X	X
	Social	Caregiver burden	Self-perceived pressure from informal care-plus^33^	Caregiver	

*Note*: This nephrology-tailored geriatric assessment, and its consensus-based development, has been described in more detail by Voorend et al.^11^

### Target Population

NGA was implemented among patients aged ≥70 years with an estimated glomerular filtration rate (eGFR) of ≤20 mL/min/1.73 m^2^ and the ability to read and understand the questionnaire. Patients were excluded in case of a history of dementia or lack of mental capacity assessed by a geriatrician.

### Implementation Strategy

**Figure 1:**
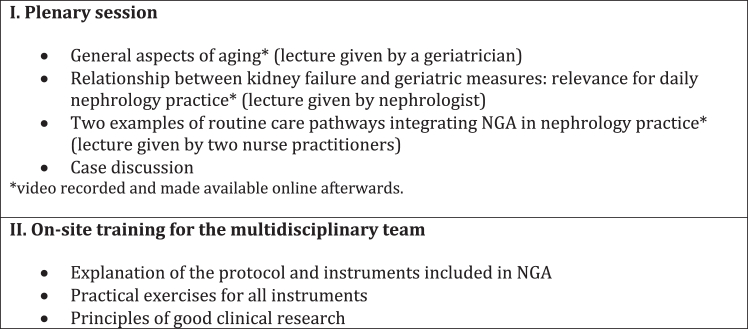
Educational program as part of the implementation strategy.

An educational program was developed to enhance awareness of geriatric impairments in the advanced CKD G4-G5 patient population and improve knowledge about geriatric assessment among involved health care professionals. The program existed of a plenary education and onsite training session, described in Figure 1. Participating centers had the flexibility to determine how they would embed the intervention in the care process. This encompassed decisions regarding who would conduct the NGA, the extent of geriatrics department’s involvement, and the organization of multidisciplinary team meetings for discussion of NGA outcomes and managing identified geriatric impairments. Additionally, optional digital entry of the patient questionnaires was offered, and a summary of NGA results was made available (after July 2020) for download after data entry in the online dashboard for data collection (see Supplementary File 1).

### Outcome

A priori, we aimed for 10 participating centers, each including 20 patients. We aimed to gain insights in variation of implementation practices, contextual changes that affected hospital participation and patient inclusion, and completeness of the NGA instruments.

Determinants of successful implementation were evaluated on 2 aspects. First, we assessed the presence of enablers and barriers to implementation and integration in work routines. A list of potential barriers and enablers was used as previously identified in a focus group study.^34^ To understand “integration in work routines” we employed the validated Dutch version of the Normalization Measure Development (NoMAD) tool,^35^^,^^36^ which has been previously used in the implementation of complex interventions in nephrology.^37^^,^^38^ Four processes of integration in work routines (ie, normalization) were assessed: sense making, cognitive participation, collective action, and reflexive monitoring (Table S1).^39^ Second, we investigated the perceived relevance of the NGA instruments to CKD care, specifically whether NGA achieved the intervention objective (enabling identification of known and unknown geriatric impairments), facilitated supportive interventions, and supported decision making for kidney failure treatment.

### Data Collection

An overview of data collection is presented in Table 2. We collected data at the hospital level using field notes to capture information on the administration of the provider-administered test set, multidisciplinary team meetings, the involved disciplines, and any contextual changes that affected hospital participation or patient inclusion. At health care provider level, a postimplementation survey was conducted among partaking health care professionals. The survey (Supplementary File 2) included questions about respondents’ characteristics, the presence of enablers and barriers to implementation, the NoMAD tool, the perceived relevance of NGA practices and its individual components, and the intervention’s ideal target group. Additionally, explanatory open-ended questions probed for deeper understanding of implementation determinants. Data were collected on the number of patient inclusions, conducted NGAs, digital patient questionnaires entries, and instrument completeness. Patient characteristics were extracted from the database managed by Nefrovisie, the Dutch quality institute for nephrology.

**Table 2:** Overview of Data Collected for Evaluation of NGA Implementation According to the Implementation Outcomes

Aim	Implementation Outcome	Data Collected	Units or Score (Range)	Source
Feasibility of implementation	Hospital participation	Number of participating hospitals that implemented NGA practices	Percentage of aimed hospitals	Field notes (hospital level)
		Implemented components of the intervention were as follows: Who executed the provider-administered test set When the provider-administered test set was done Outcomes discussed in multidisciplinary team meetings Who attended multidisciplinary team meetings Contextual changes that affected hospital participation and patient inclusion	Discipline Single or multiple visit Yes/no Disciplines -	
	Patient inclusion	Number of patient participants Patient characteristics	Count Sex Age eGFR	Database collection (patient level)
		Completeness of the NGA (ie, provider-administered test sets and patient questionnaire) Completeness of items of each NGA instrument	Percentage completed test sets and questionnaires Percentage complete instruments	
Determinants of (un)successful implementation	Enablers and barriers to implementation	Rating of the presence of potential barriers and enablers identified in a preceding study^34^ Respondents’ 3 perceived most important enablers and barriers for successful implementation in their center.	10-point Likert presence scale Top 3 given by respondent	Postimplementation survey among partaking health care professionals
	Integration in work routines	NoMAD tool: Three general normalization items on the past, current, and likelihood of future use of the intervention Eighteen questions were used to measure the normalization subconstructs	0-10 visual analog scale; higher scores indicating more use. 5-point Likert scale (1-disagree to 5-agree).	
	Perceived relevance of the NGA	Agreement of relevance of NGA practices as a whole: Rating 3 aspects of the intervention objective, ie, NGA supports in the following: 1. Identification and objectivities of impairments 2. Adjusting or supplementing current treatment strategies 3. Informing future treatment decision for kidney replacement therapy	10-point Likert scale: 0-“strongly disagree” to 10-“strongly agree”	
		Rating all 17 individual NGA instruments for their relevance to the intervention objective (see above)	5-point Likert scale: 1 (not at all relevant) to 5 (very relevant)	
		Appraisal of the target group by rating: 1. Age limit (70+ y) 2. Kidney function limit (eGFR < 20 mL/min/1.73 m^2^)	3 options: “too extended,” “sufficient,” or “too narrow”	
	Explanatory qualitative information	Understanding on determinants of implementation	Open questions	
Additional information		Survey respondents’ characteristics	discipline, years’ experience in current function and department, affiliated hospital	

Abbreviations: eGFR, estimated glomerular filtration rate; NGA, nephrology-tailored geriatric assessment; NoMAD, Normalization MeAsure Development.

### Survey Sampling

Participants for the postimplementation survey were purposively sampled by asking each center’s contact person to list at least 5 colleagues from various disciplines involved in the care of older patients with CKD. The survey was disseminated after study inclusion closed in May 2021. Eighty-one professionals received a personal email invitation to participate in an online survey using Qualtrics software (Qualtrics, version 2021). Nonresponders received reminders 2 weeks later. We assured confidentiality through pseudonymized analyses of the data.

### Data Analysis

Feasibility of implementation was assessed by analyzing per center the implemented components of the intervention, patient inclusion and completeness of NGA instruments. Demographic and clinical patient data, along with survey respondent characteristics and quantitative survey responses, were presented descriptively. To assess integration into routine work (ie, normalization), we used paired *t* tests to compare current and anticipated future use of NGA. Average construct scores were calculated for each participant, excluding “nonapplicable” items. Higher scores signified better-perceived implementation.^37^^,^^39^ A mean subconstruct score <4 indicated potential for improvement. The overall perceived relevance of the NGA set was appraised by the sample mean of the respondents’ judgment on 3 intervention objective achievement questions. A score of ≥7 was considered relevant (ie, successful in reaching the intervention objective). For the specific instruments, we strived for a rating of ≥4 (ie, “relevant” or “very relevant”) among 70% of the respondents. IBM SPSS statistics for Windows (version 25) was used for quantitative analysis.

Explanatory qualitative data analysis involved open-ended survey responses, initially coded by 2 authors independently (ie, project lead [CV] and an uninvolved author [LB]). Codes were discussed to ensure agreement on the final interpretations. We did not use qualitative research software. Triangulation of the quantitative and qualitative data was done by exploring (dis)agreements within and between hospitals in the survey responses and participants or centers with notable normalization levels.

The Revised Standards for Quality Improvement Reporting Excellence (SQUIRE 2.0)^40^ and Consensus-Based Checklist for Reporting of Survey Studies guided reporting of our results.^41^

### Ethical Considerations

The POLDER study’s protocol was approved by the Medical Research Ethics Committee Leiden-Den Haag-Delft (reference NL65322.098.18) to facilitate patient data collection and analyses, and the study was conducted according to the principles of the Declaration of Helsinki and the Dutch Medical Research Involving Human Subjects Act. The POLDER study is registered in the Netherlands Trial Registry (trialsearch.who.int: NTR7310).

## Results

### Hospital Participation

Ten of the 16 initially approached centers implemented NGA practices, while 5 declined because of a lack of reimbursement and personnel shortages. Two nephrology units independently agreed to participate but later merged into one. Implementation started between October 2018 and March 2020, with the study start and inclusion period extended because of logistic and COVID-19–related challenges, and patient inclusion concluded in April 2021.

Geriatric tests were conducted by a nephrology nurse (practitioner) (N = 6 centers, 60%), a geriatrician or geriatric nurse (N = 3, 30%), or a research nurse (N = 1, 10%), see Table S2. Visits for the geriatric tests were combined with regular nephrology appointments at most centers (N = 9, 90%). Patient questionnaires were sent in advance of their visit for at-home completion or given afterward. Approximately 15% (n = 28) of patients digitally entered their questionnaires; this was done at 7 centers (ranging from 8%-38% per center). At 9 centers, outcomes were discussed in multidisciplinary team meetings with geriatric expertise involved in 7 settings. One center conducted meetings if geriatric impairments that needed further involvement of a geriatrician were detected.

### Patient Inclusion and Characteristics

A total of 194 patients gave informed consent, with 3 unable to participate because of deteriorating health status (n = 2, 1%) or no-show (n = 1, 0.1%). NGA was performed in 191 patients. Participation varied between n = 10 and n = 30 per hospital. Most participants were men (n = 135, 71%). Median age was 77.5 (interquartile range, 74.3-81.9) years, and mean eGFR at the time of NGA was 15.0 ± 4.4 (SD) mL/min/1.73 m^2^.

### Completeness of NGA

In total, 187 patients (98%) returned the patient questionnaire. All but one patient (n = 190, 99%) finalized the provider-administered test set. Table S3 reports completeness of the NGA instruments.

Somatic instruments were completed by all patients. Over 90% of the patients completed measures for activities of daily living, fall risk, 6-item Cognitive Impairment Test, Montreal Cognitive Assessment (MoCA), Letter Digit Substitution Test, and depressive mood. Measures of caregiver burden and nutritional status were conducted in 64% (n = 121) and 82% (n = 156) of the patients and were less often completed (60% and 66%, respectively).

### Survey Respondents’ Characteristics

Of 81 invited health care professionals, 71 responded (88% response rate). Table 3 summarize the survey respondent characteristics, with a median of 6 professionals (range, 5-12) participating per hospital.

**Table 3:** Survey Respondents’ Characteristics and Outcomes

Survey Participant Characteristics	n = 71
Respondents per hospital, median (range)	6 (5-12)
Working experience (y), median (range)	
in current profession	9 (1-30)
in current department	8 (1-39)
Clinical role, n (%)	
Nephrologist	27 (38)
Geriatrician	10 (14)
Nurse practitioner^a^	10 (14)
Nurse (nephrology)	11 (16)
Nurse (geriatrics/geriatrics-nephrology)	2 (3)
Physician assistant (nephrology)	1 (1)
Social worker	7 (10)
Dietitian	2 (3)
Research nurse	1 (1)

a Including one respondent in training.

### Enablers and Barriers to Implementation

The most frequently cited top 3 enablers for successful implementation included collaboration with geriatric department (n = 45, 76%), multidisciplinary meetings and reports (n = 39, 66%), assessment performed by nurses (practitioners) (n = 26, 44%), and discussion of purpose and outcomes of the test with patients (n = 26, 44%). Common barriers to implementation included patient illiteracy or non-Dutch speaking (46% of respondents’ top 3 barriers, n = 25), lack of time (n = 20, 37%), burden for patients (n = 20, 37%), and lack of patients’ willingness or eagerness to participate in NGA (n = 17, 31%). The open-ended questions showed concerns on patient burden: difficulty of NGA questions (Table S4, Q41), the toll (ie, time and burden) of hospital visits (Table S4, Q42), and the negative connotation of “geriatric assessment” (Table S4, Q43 and Q44). Table 4 presents the respondents’ agreement on the presence of implementation enablers and barriers. Interestingly, respondents reported that patients were often willing to participate in NGA (mean score as enabler, 6.9/10 ± 1.68), although this was frequently cited as a top 3 barrier.

**Table 4:** Presence of Enablers and Barriers of Implementation of the NGA and Those Most Frequently Rated for Successful Implementation

	The Following Reasons May Have Led to Good or Less Good Implementation. In Your Experience, Were These Reasons Present In Your Hospital’s NGA Performance?	N	Agreement, mean ± SD		Presence	
	*Enablers*			Totally disagree (1)		Totally agree (10)
**☺**	The purpose and results of the tests were discussed in detail with the patient	48	6.8 (2.09)			
☹	Patients were willing and available for the geriatric assessment	56	6.9 (1.68)			
**☺**	Good cooperation with geriatrics department	58	8.1 (1.79)			
**☺**	Multidisciplinary consultation and reports in which NGA outcomes and treatment policy were discussed	65	7.7 (2.03)			
	Support from other disciplines (eg, dietitian, social worker) in the administration and interpretation of NGA	62	6.6 (2.77)			
**☺**	Suitable (and trained) personnel were sufficiently available to administer the NGA	53	7.2 (1.91)			
	The outpatient schedule was easy to adjust for NGA administration	50	6.0 (2.04)			
	Management supports the implementation of the NGA	51	6.9 (2.22)			
	The sum-score forms in the dashboard (available from July 2020) were helpful	28	6.1 (2.81)			
	*Barriers*					
☹	The NGA is too much of a burden for many patients	55	4.8 (1.81)			
☹	NGA was performed to a limited extent because many patients had low health literacy or because of a language barrier	50	4.5 (1.88)			
	Reluctance in the Nephrology department to involve geriatrics/elderly care in routine care	61	3.3 (2.35)			
	Loss of geriatric knowledge and practical skills (for example because of team changes)	47	3.3 (2.25)			
☹	Time constraints restricted carrying out the NGA	46	4.7 (2.32)			
	Lack of budget is a reason to carry out NGA less often or adequately	39	3.6 (2.28)			
	

*Notes:* Both enablers and barriers are sorted by patient-related, multidisciplinary cooperation, and organizational aspects. Score range: 1 (totally disagree) to 10 (totally agree). Four enablers (☺) and 4 barriers (☹) that were most frequently in respondents’ top 3 for successful implementation. One enabler was mentioned as a determinant in the top 3 barriers for implementation.

Abbreviations: NGA, nephrology-tailored geriatric assessment.

### Integration Into Work Routines

**Figure 2:**
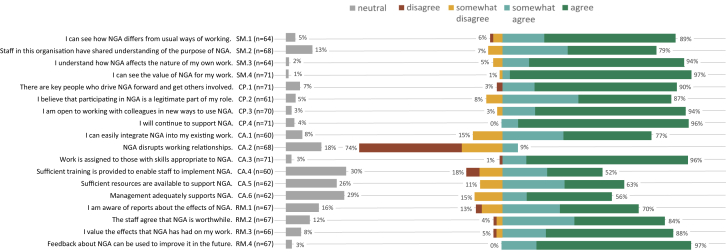
Response to statements on normalization of NGA. Figure bars show the percentage of respondents reporting their agreement with the NoMAD statements. The number of respondents lower than n = 71 bypassed the statement by indicating “not relevant for my role.” Questions relate to the constructs of the NPT framework, ie, SM: sense making, CP: cognitive participation, CA: collective action, and RM: reflexive monitoring. Abbreviations: NGA, nephrology-tailored geriatric assessment; NoMAD, Normalization measure development; NPT, normalization process theory.

The general NoMAD questions indicated that NGA was not fully embedded at the time of the survey (mean, 6.9/10 ± 2.01 versus mean, 6.7 ± 1.42; *P* < 0.001). Figure 2 presents responses to each of the NoMAD questions. Although 97% (n = 69) recognized the potential value of NGA and 96% (n = 49) continued to support it, there were opportunities to enhance integration, according to 97% (n = 65). Table S4 and Figure S1 show the mean NoMAD (sub-)construct scores and qualitative analysis of the open-ended survey questions. Respondents found NGA meaningful (Sense Making mean score, 4.6 ± 0.59) and were committed to making it work (Cognitive Participation mean score, 4.6 ± 0.50). To a somewhat lesser extent, the respondents acknowledged the efforts of working together to make NGA work (Collective Action mean score, 4.1 ± 0.66) and appraised the effect of NGA (Reflexive Monitoring mean score, 4.3 ± 0.64). Concurrently, respondents recognized the potential improvement of sufficient training, resources, and knowledge about the effects of NGA, indicated by mean scores of <4 for the subconstructs of skill set workability, contextual integration, and systemization (Figure S1). The open-ended questions showed that nurses’ lack of time and availability hampered integration in routine work (Table S3, Q12), and suggested improvements such as reducing the number of tests (Table S3, Q36-Q39), and improvement of interdisciplinary cooperation (Table S3, Q28 and Q29).

### Perceived Relevance of the NGA Instruments to Care of Patients With CKD

Health care professionals perceived NGA instruments as successful in reaching the intervention objective and its aimed effects, with an overall score of 7.8/10 ± 1.16. The relevance of each included instrument is shown quantitatively in Table 5 and qualitatively in Table S5. Providers rated the relevance of the MoCA and the Clinical Frailty Score as high (mean, 4.5/5 ± 0.60 and mean, 4.4 ± 0.69, respectively), while they appraised polypharmacy and the Life Orientation Test as less relevant (mean, 3.5 ± 0.98 and mean, 3.6 ± 0.79, respectively). Less than 70% of the users were convinced of the relevance of the latter 2 instruments and handgrip strength, Letter Digit Substitution Test, and the Patient Generated-Subjective Global Assessment.

**Table 5:** Appraisal of Relevance of the Instruments Included in the NGA

Domain	Instrument	Respondents (n)	Survey Outcomes				
			Relevance score^a^ mean (SD)	Not relevant n (%)	Neutral n (%)	Relevant n (%)	
Functional status	Activities of daily living (Katz ADL-6)	59	4.2 (0.70)	0 (0%)	9 (15%)	50 (85%)	
	Instrumental Activities of daily living (Lawton)	59	4.1 (0.70)	0 (0%)	11 (19%)	48 (81%)	
	Handgrip strength	60	3.7 (0.88)	3 (5%)	21 (35%)	36 (60%)	
	Fall risk assessment	61	4.1 (0.68)	1 (2%)	9 (15%)	51 (84%)	
Cognitive functioning	Montreal Cognitive Assessment	61	4.5 (0.60)	0 (0%)	3 (5%)	58 (95%)	
	6-item Cognitive Impairment Test	59	4.1 (0.80)	1 (2%)	13 (22%)	45 (76%)	
	Letter Digit Substitution Test	56	3.7 (0.90)	5 (9%)	14 (25%)	37 (66%)	
Psychological status/mood	Whooley-questions/Geriatric Depression Scale-15	60	4.2 (0.81)	1 (2%)	7 (12%)	52 (87%)	
	Life Orientation Test-Revised	59	3.6 (0.79)	3 (5%)	28 (47%)	28 (47%)	
PROM’s	HRQoL: 12-item Short Form Health Survey	62	4.1 (0.69)	0 (0%)	11 (18%)	51 (82%)	
	Dialysis Symptom Index	59	4.0 (0.73)	0 (0%)	15 (25%)	44 (75%)	
Somatic status	Surprise question	63	4.0 (0.81)	2 (3%)	15 (24%)	46 (73%)	
	Clinical Frailty Score,	64	4.4 (0.69)	1 (2%)	4 (6%)	59 (92%)	
	Charlson Comorbidity Index	60	4.0 (0.80)	3 (5%)	10 (17%)	47 (78%)	
	Polypharmacy	60	3.5 (0.98)	10 (17%)	21 (35%)	29 (48%)	
Nutrition	Patient-Generated Subjective Global Assessment	62	3.8 (0.88)	4 (6%)	15 (24%)	43 (69%)	
Social	Caregiver burden: SPICC-plus	64	4.0 (0.79)	4 (6%)	9 (14%)	51 (80%)	
	

*Note:* The percentages in the fifth to seventh columns do not add up to exactly 100% because of rounding differences.

Abbreviations: HRQoL, health-related quality of life; NGA, nephrology-tailored geriatric assessment; PROM, patient-reported outcome measure; SPICC, self-perceived pressure from informal care.

a Scoring was done on a 5-point Likert scale: 1 (not at all relevant) to 5 (very relevant).

### Perceived Relevance to the Target Group

Survey respondents had varying opinions on age and kidney function cutoffs for NGA practices. Cutoffs of >70 years and eGFR of <20 mL/min/1.73 m^2^ were too narrow for 24% (n = 16) and 18% (n = 12), good for 67% (n = 44) and 69% (n = 45), and too broad for 9% (n = 6) and 12% (n =8), respectively. Several respondents qualitatively addressed that NGA should be tailored to each patient’s situation, considering the progression of CKD, clinical judgment, and biological age rather than calendar age or kidney function.

## Discussion

Our findings demonstrate the feasibility of implementing NGA in older patients with advanced CKD. Furthermore, they offer recommendations for extending guidance on how to integrate geriatric assessment in nephrology care. We identified determinants of successful implementation and barriers. Collaborative involvement from the geriatrics department, nurse practitioners, and multidisciplinary meetings enhanced implementation, while limited collective action, lack of time and personnel, and patient-related factors, such as burden and low literacy, hampered it. Integration of NGA into work routines could be enhanced because it was not yet fully integrated. Nonetheless, health care professionals recognized its relevance in care of patients with CKD.

Our findings contribute to the emergence of models for integrating geriatric care into hospital practices,^42^^,^^43^ gaining recognition for geriatric assessment as a predictive and rehabilitative instrument for older patients across various medical disciplines. Compared with oncology^42^ and acute care patients, care of patients with CKD involves longer-standing patient–provider relationships and generally longer-lasting decision trajectories. However, impairments, primarily cognitive problems, often go unnoticed in patients with CKD.^3^ Therefore, geriatric assessment should focus on detecting and managing such impairments and also on adapting how education and information on treatment selection is delivered. Similar to other medical fields, substantial implementation barriers were recognized, mainly concerning practicalities and resources such as time constraints and unavailability of health care personnel.^42^^,^^44^^,^^45^

Our study provides a practical example of the previously recognized need to integrate standardized geriatric assessment into routine nephrology practice.^3^^,^^46^, ^47^, ^48^, ^49^, ^50^ Our insights extend the paucity of guidance on the incorporation of geriatric assessment in nephrology care.^13^^,^^51^ Predominantly, collaboration with the geriatric department appears to be essential because it provides expertise for advice and referrals for management of identified impairments.^13^^,^^15^^,^^16^ Although the extent of geriatrics involvement may vary from overseeing all NGA practices to availability for referrals only, both are viable approaches.^13^

To improve integration of NGA in work routines, our results showed that collective action (ie, how people work together to make NGA practices work) deserves attention. Our study found that implementation often relied on one or a few key persons, which makes maintenance of NGA practices vulnerable. Evidence regarding the benefits and use of NGA would help to improve collective action. Related to the latter, discussion of carefully summarized results of NGA in multidisciplinary team meeting (including geriatric health care professionals) is essential.^34^^,^^44^^,^^52^ This will help to create awareness on the presence and relevance of detected cognitive and functional impairments and implications for treatment and supportive care.

A potential facilitator for implementation into routine nephrology care may be the integration of summarized NGA outcomes in electronic patient files. This may increase multidisciplinary use of recurring geriatric assessment and support its interpretation. In oncology, a tailored geriatric assessment summary with guided recommendations has facilitated and improved patient care,^53^ and web-based applications for geriatric assessment seem promising.^54^^,^^55^ Involvement of older (CKD) patients in future research and development of digital tools is desired.^56^

The negative effect of patient burden on implementation was noted, as in previous studies.^34^^,^^42^^,^^57^ Therefore, it is essential to integrate NGA in regular nephrology hospital visits and limit the frequency and duration of prolonged hospital visits. Adjustments to NGA practices for individual cases may be necessary and helpful, whereas refinement of the assessment by modifying the test set can also improve acceptance and implementation.

In absence of a gold standard,^58^ our test set was derived in a foregoing pragmatic consensus trajectory.^11^ If time for assessment practices is limited, some instruments could be considered for omission or substituted with shorter instruments, preferably only after the discriminative and predictive value of the tests is investigated in the CKD stage G4-G5 population. For example, polypharmacy was irrelevant to a substantial part of our NGA users, potentially because of overlap with repeated attention to this topic in routine medical care. The instrument on nutrition (Patient Generated-Subjective Global Assessment) showed considerable incompleteness and perceived irrelevance in our study, potentially because of inconvenient usage and overlapping practices of dieticians who already have a clearly defined role in multidisciplinary management of patients with CKD in routine care. In addition, the Life Orientation Test, Letter Digit Substitution Test, and handgrip strength were perceived less relevant to the professionals. Based on our evaluation and other literature,^15^^,^^59^, ^60^, ^61^, ^62^ both the Clinical Frailty Score^30^ and MoCA^21^ are a relatively new to outpatient nephrology care but may be promising instruments. The caregiver burden (Self-Perceived Pressure from Informal Care) questionnaire, though perceived relevant to professionals, reported considerable incompletion, which was to a large extent explained by those patients that did not need caregiver support.

Further development of NGA should focus on inclusiveness by incorporating linguistic and cultural factors.^34^^,^^42^^,^^57^ Although illiteracy was an exclusion criterion for the present study, apparently, health care professionals determined that assessment was still too elaborate for some patients. In our view, low health literacy as a barrier to implementation of geriatric assessment has been underreported in scientific studies.

Flexibility in adapting the abovementioned fixed parts of NGA according to organizational circumstances, resource constraints, and patient profile has proven advantageous for successful implementation.^45^^,^^63^

A strength of our study is that we are among the first to explore and report multicenter implementation of geriatric assessment in routine CKD care quantitatively, enriched by an explanatory qualitative analysis. Although NGA implementation was feasible for hospital participation, we fell short of our patient inclusion goal in half of the centers. This was due to incomplete integration of NGA practices, the effect of the COVID-19 pandemic, and scientific study formalities. However, the subsequent DIALOGICA (Dialysis or Not: Outcomes in Older Kidney Patients With Geriatric Assessment) study^64^ now involves 35 centers and >600 patients, showcasing widespread feasibility.^65^ Limitations of our study are that we did not structurally collect data on the management plan of identified geriatric impairments (eg, outcomes of multidisciplinary team meetings and follow-up of patients). We also did not include an economic component or use a randomized controlled or pre- or postsurvey design. This prevents us from drawing conclusions regarding efficacy, effectiveness, and efficiency of NGA practices. Furthermore, survey responders were committed to this topic, which may have led to bias toward positive outcomes. However, no financial incentives or inclusion fees were provided. Activities were organized in daily practices. Although some hospitals made use of local research departments for data entry and study-related logistics, this was outside the scope of the current implementation study but may have influenced implementation.

Future research should evaluate implementation of management of geriatric impairments and establish the efficacy of geriatric practices in nephrology,^66^ as was done in other medical fields.^67^^,^^68^ Also, the acceptability from patient perspective^34^ and the use of cross-cultural instruments^57^ needs further investigation.

In conclusion, geriatric assessment could successfully be integrated in CKD care and is perceived as relevant by partaking health care professionals.

## Supplementary Materials

Supplementary File (PDF) Figure S1; Item S1-S2; Tables S1-S5.
