# Supplementary material for: Geriatric Assessment in CKD Care: An Implementation Study
Source: Kidney Med. 2024 Mar 21;6(5):100809. doi: 10.1016/j.xkme.2024.100809 (PMC11039322; doi:10.1016/j.xkme.2024.100809)
Supplement: Supplementary File (PDF) — Figure S1; Item S1-S2; Tables S1-S5. [file mmc1.pdf]

Respondents stated that:

Reconfiguation: Some parts of NGA may not fit all patients, need for adjustments for illiterate non-Dutch speaking persons were mentioned. Further development of NGA is needed said some; e.g. through multidisciplinary team meetings, availability of geriatrics, potential use of shorter screening tool for full NGA.

Individual appraisal: Most respondents value the effects of NGA; i.e. holistic view on frailty, improved patient-conversations and personalized care. But some noted to see no clear effect of NGA.

Communal appraisal: More demand for NGA is noted, multidisciplinary cooperation improved due to NGA. But the value is not seen by everyone; mostly due to needed time-investments, burden for patients, or lack of concrete outcomes.

Systemisation: Some noted that effectiveness of NGA has not been established. According to others effectiveness is sufficiently proven in medical fields (e.g. oncology) or from own experience.

Contextual integration: Management mostly supports NGA but not in every centre. Lack of resources (i.e. time, availability of (dialysis) nurses, and budget for multidisciplinary cooperation) was noticed frequently. Information dissemination could be optimized, e.g. by software.

Skill set workability: Lack of knowledge and experience with NGA may hamper communal appraisal. Some said that inter-hospital knowledge exchange and (continued) training is needed, e.g. for low illiterate patients.

Relational integration: Confusion on focal point or specialism in lead may disturb working relationships. Multidisciplinary collaboration, involvement and communication is needed, and is sometimes subject for improvement. Multidisciplinary team meetings were acknowledged.

Interactional workability: Although some stated that NGA fits well in existing outpatient care (through either standard geriatrics visit or integrated in nephrology visit with reference to geriatrics if needed), some centres needed to ask a research nurse and it was noted that not all patients were screened.

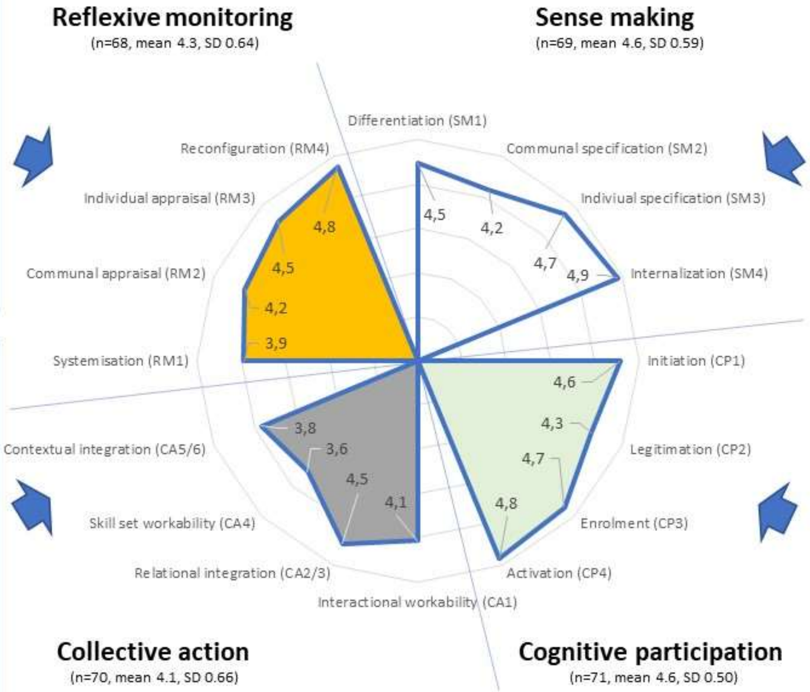

Respondents stated that:

Differentiation: NGA differs from usual care in providing objective insights, identification of unnoticed impairments. This leads to better conversations on suitable care (support) between HCW and patients, and among HCWs.

Communal specification: In some centres the goal of NGA was widely acknowledged, but not everywhere.

Individual specification: NGA is informative for patients' decision making on KRT e.g., to dispel doubts. Although a few respondents have doubts on the relevance of NGA and its value for the decision-making process for all patients.

Internalization: NGA is of value for consideration of treatment options, to emphasize quality of life and provide suitable care. A minority of the respondents do not think NGA is of value; as it would not lead to concrete advices and only sporadically contributes to the decision-making process.

Initiation: Key persons are those who execute the NGA and maintain the connection between nephrology and geriatrics; i.e. nephrology nurse (practitioner) with geriatric affinity, sometimes a nephrologist, or a geriatric nurse or -specialist. It is of risk that this often relies on one person.

Legitimation: while one respondent described that NGA is not for all CKD patients considered legitimate; another stated that the increasing number of frail older CKD patients legitimates standardized NGA.

Enrolment: working together with the geriatrics nurses or specialists is acknowledged.

Activation: some respondents have controversy to assess NGA for all patients, but majority stated to support NGA, for reasons that it provides HWC a holistic view on patients' status, is useful to support treatment and improves care.

Figure S1: Integration of NGA in routine care: visualization of the mixed methods approach using of normalization construct scores and open-ended questions. The petal charts presents the average score for each NPT subconstruct of normalization on a Likert scale of 1 (strongly disagree) to 5 (strongly agree). The boxes describe the determinants of NGA integration in routine work as derived from the qualitative analysis of open-ended questions. Supporting evidence (quotes) are presented in Supplemental Table S2.

Abbreviations: HCP, health care professionals; NGA, nephrology-tailored geriatric assessment; KRT, kidney replacement therapy.

POLDER

Item S1: Visualization of Summary of Individuals' NGA Results

Geriatric assessment scores

Below you will find an overview of the scores of the professional-administered tests used in the POLDER study. Please note, the explanation in the third column (interpretation) is not intended as a clinical cut-off point for diagnosis or the use of interventions. Always take into account the overall picture and clinical judgment.

Patient: 9002, female, 81 jaar (+/- 1 jaar), education level: low

Inclusion date: 12-12-2020

Measurement: baseline (date: 16-2-2021)

Clinical judgement	Score	Score range (interpretation)
Surprise question	1x ja / 3x nee	
Clinical frailty scale	6 (moderate frailty)	1 - 9 (1=very fit, 9=terminally ill)
**Comorbidity**		
Charlson score (not age-adjusted)	6	0 - 33
**Mental functioning**		
MOCA score	26	0 - 30 (≥26 is considered not impaired) [1]
Six Item Cognitive Impairment Test	10	0 - 28 (≥11 suggest moderate to severe impairment of cognitive functioning) [2]
Letter digit substitution test Correct after 60 sec	25 (=p76)	Percentile based on age, gender and education level [3]
Depression / mood		
GDS-2/Whooley questions, count 'yes'	1	0 - 2 (if score is 0, GDS-15 is not assessed)
GDS-15 score	6	0 - 15 (≥6 possible depression) [4]
**Physical functioning**		
Maximum handgrip strength	32 (=p90)	0-60 kg, higher indicates more strength. Percentile based on age and gender [5]
Fall history	Yes	Past year ≥1 times fallen
Fall anxiety score	8	1-10 (1=no fear, 10=very afraid)
**Nutritional status (PG-SGA)**		
Total worksheet 1 to 4	24	0-52 (≥9 possible nutritional interventions needed) [6]
Global staging	A (well nourished)	Stage A (well nourished)<br>Stage B (moderately malnourished)<br>Stage C (severely malnourished)

References: 1) MOCA: Nasreddine et.al. (2005, J Am Geriatr Soc), 2) 6-CIT: Van Tuijl et al. (2002, Int J Ger Psych), 3) LDST: Van der Elst et al. (2006, J Clin Exp Neuropsychol), 4) GDS-15: Dennis et al. (2012, Age Ageing), 5) Handgrip strenght: Dodds et al. (2014, Plos One), 6) PG-SGA: Koster et al. (2020, Nutrition)

** This form has been translated from Dutch, and added as supplementary file 1 to Voorend et al., 2023 Implementation of geriatric assessment in CKD care; a quality improvement initiative.*

POLDER

Patient questionnaire scores

Below you will find an overview of the scores from the patient questionnaire of the POLDER study.

Study id: 9002
Inclusion date: 12-12-2020
Measurement: baseline (date: 16-2-2021)

Heath-related quality of life	Score	Score range (interpretation)
SF12 – physical component	40,4	Higher score indicates better well-being (50,3 general Dutch population [1] 35,6 dialysis patients [2])
SF12 – mental component	41,6	Higher score indicates better well-being (52,9 general Dutch population [1] 47,7 dialysis patients [2])
DSI - Dialysis Symptom Index: count 'yes'	6 symptoms	See specific symptoms on next page
**Mental functioning**		
Optimism (Life-orientation test)	Not calculated due to missing items	0-24 (higher scores indicates higher optimism, average score general population: 16.2 [3])
**Physical functioning**		
Katz-ADL6	2, somewhat dependent (≥2)	0-6 (≥2: somewhat, ≥4: moderately, 6: complete dependent)
IADL-Lawton	5	0-8 for females (higher score is less dependent)
**Nutritional status (PG-SGA)**		
Nutritional status (patient part)	8	0-36 (≥ 6 indicates malnutrition) 4
**Caregiver burden**		
Caregiver burden (SPICC-plus)	2, minor burden (1-3)	0-15 (0: no burden (21%),<br>1-3: minor burden (33%),<br>4-8: moderate burden (27%),<br>9-15: severe burden (19%) 5)

References:

1) CBS Statistics Netherlands (2009)
2) Pilot PROMs-NNL, Nefrovisie (2017)
3) Optimism (LOT-R): Hinz et al. (2017, J Clin Health Psychol)
4) PG-SGA-SF: Kosters et al. (2020, Nutrition)
5) SPICC-plus: De Boer et al. (2012, Tijdschr Gerontol Geriatr)

Symptoms experienced in past week		Symptom presence, burden
1. Constipation		No
2. Nausea		No
3. Vomiting		No
4. Diarrhea		No
5. Decreased appetite		No
6. Muscle cramps		No
7. Swelling in legs		No
8. Lightheadedness or dizziness		No
9. Shortness of breath		No
10. Restless legs or difficulty keeping legs still		No
11. Numbness or tingling in feet		Yes, a little bit
12. Feeling tired or lack of energy		No
13. Cough		No
14. Dry mouth		No
15. Bone or joint pain		No
16. Chest pain		No
17. Headache		No
18. Muscle soreness		No
19. Difficulty concentrating		No
20. Dry skin		No
21. Itching		No
22. Worrying		No
23. Feeling nervous		No
24. Trouble falling asleep		No
25. Trouble staying asleep		No
26. Feeling irritable		Yes, a little bit
27. Feeling sad		Yes, a little bit
28. Felling anxious		Yes, somewhat
29.Descreased interest in sex		Yes, a little bit
30. Difficulty becoming sexually aroused		Yes, a little bit
Other symptom 1:		
Other symptom 2:		
Other symptom 3:		

Item S2: Post-implementation survey amongst health care professionals
Translated from Dutch.

Polder Evaluation: Implementation NGA

Start of Block: Consent

Consent Form

Thank you for participating in the evaluation of the nephro-geriatric assessment (NGA) in the POLDER study. Your opinion on this innovation in kidney failure care is essential!

This POLDER sub-study aims to:

1. Evaluate the implementation of nephro-geriatric assessment (NGA) in routine care for patients with kidney failure
2. Improve the test set.

The questionnaire consists of 6 parts: *integration of NGA in routine care (using the validated NoMAD questionnaire), contribution of NGA to routine care, reasons for successful or limited implementation, evaluation of the specific instruments in the NGA, improvements for NGA practices, and general data.*

It takes about 15 minutes to complete the multiple choice questions and scoring statements. Optionally, you can provide us additional explanations. We process your data in such a way that the answers cannot be traced back to you personally. This applies to both the report to the funder (Kidney Foundation) and the scientific article.

Would you like to have more information? Please see full information letter at our website: *www.polderstudie.nl/nl/over-polder/evaluatie*

Do you give permission for the use of your answers for this research and their (scientific) publication?

○ Yes, I give permission

○ No, I don't give permission < end of questionnaire>

Item S2: Post-implementation survey amongst health care professionals
Translated from Dutch.

Explanation:

Definition Nephro-Geriatric Assessment (NGA):
he questionnaire is about 'embedding NGA in routine care’. This includes the patient questionnaire and the professional-administered test set of POLDER study, and using the results of these tests in CKD-care.

The NGA test set comprised of different domains and instruments:

- physical functioning: hand grip strenght, (instrumental) Activities of Daily Living, risk of falling,
- cognitive functioning: MoCA, 6-CIT, Letter Digit Substitution Test,
- mood: geriatric depression scale, optimism,
- somatic: Clinical Frailty Scale, Charlson Comorbidy Index, polypharmacy,
- nutritional status: PG-SGA,
- PROMs: quality of life, symptoms,
- caregiver burden: EDIZ-plus.

Assessment may have been done with interference of the geriatrician. Ideally, the results should be discussed with the patient in a multidisciplinary consultation.

End of Block: Consent

Item S2: Post-implementation survey amongst health care professionals
Translated from Dutch.

Start of Block A: General data

Q1 What is your profession?

- ○ Nephrologist
- ○ Geriatrician / internist-geriatrician
- ○ Nurse practitioner, discipline: ____
- ○ Nephrology nurse
- ○ Geriatric nurse
- ○ Dietician
- ○ Social worker
- ○ Research nurse
- ○ Other, please specify: ____

Q2 How many years of work experience do you have in this position?

____

Q3 How many years have you been working for this department in this hospital?

____

Q4 Through which hospital are you connected to POLDER?

<multiple choice answer option for all 10 hospitals>

Q5 Have you been directly involved in the design and/or implementation of the NGA in your hospital?

Yes / No

End of Block A: General data

Start of Block B: Integration in routine care (NoMAD questionnaire)

Item S2: Post-implementation survey amongst health care professionals
Translated from Dutch.

Q6 When you use the nephro-geriatric assessment, how familiar does it feel?

Not at all familiar — Neutral — Feels completely familiar

(0) (1) (2) (3) (4) (5) (6) (7) (8) (9) (10)

Q7 Do you feel the nephro-geriatric assessment is currently a normal part of your work in care for older CKD patients?

No, not at all — Neutral — Yes, completely

(0) (1) (2) (3) (4) (5) (6) (7) (8) (9) (10)

Q8 Do you feel the nephro-geriatric assessment will become a normal part of your work in care older CKD patients?

No, not at all — Neutral — Yes, completely

(0) (1) (2) (3) (4) (5) (6) (7) (8) (9) (10)

Q10 For each statement please select an answer that best suits your experience. If the statement is not relevant to you please select *'not relevant to my role'*.

	Disagree (1)	Somewhat disagree (2)	Neither agree nor disagree (3)	Somewhat agree (4)	Agree (5)	Not relevant to my role
1. I can see how the NGA differs from usual ways of working in the kidney failure outpatient clinic.	○	○	○	○	○	☐
2. Staff in this organization have a shared understanding of the purpose of NGA.	○	○	○	○	○	☐
3. I understand how the nephro-geriatric assessment affects the nature of my work at the kidney failure outpatient clinic.	○	○	○	○	○	☐
4. I can see the potential value of the nephro-geriatric assessment for my work in older CKD patient care.	○	○	○	○	○	☐

Q11 Could you briefly elaborate your answers? (optional)

____

____

Item S2: Post-implementation survey amongst health care professionals
Translated from Dutch.

Q12

	Disagree (1)	Somewhat disagree (2)	Neither agree nor disagree (3)	Somewhat agree (4)	Agree (5)	Not relevant to my role
5. There are key people who drive the NGA forward and get others involved.	○	○	○	○	○	☐
6. I believe that participating in the NGA is a legitimate part of my role.	○	○	○	○	○	☐
7. I'm open to working with collegues in new ways to use the NGA.	○	○	○	○	○	☐
8. I will continue to support the NGA.	○	○	○	○	○	☐

Q13 Could you briefly elaborate your answers? (optional)

Who are the key people?
Why do you support the NGA, or not?
What is your motivation for performing the NGA?

____

____

Item S2: Post-implementation survey amongst health care professionals
Translated from Dutch.

Q14

	Disagree (1)	Somewhat disagree (2)	Neither agree nor disagree (3)	Somewhat agree (4)	Agree (5)	Not relevant to my role
9. I can easily integrate the NGA into my existing work.	○	○	○	○	○	☐
10. The NGA disrupts working relationships.	○	○	○	○	○	☐
11. Work is assigned to those with skills appropriate to the NGA.	○	○	○	○	○	☐
12. Sufficient training is provided to staff to implement NGA.	○	○	○	○	○	☐
13. Sufficient resources are available to support the NGA.	○	○	○	○	○	☐
14. The management adequately supports NGA.	○	○	○	○	○	☐

Q15 Could you briefly elaborate your answers? (optional)

If the nephro-geriatric assessment is not easy to integrate; Why not?
Why does NGA disrupt working relationships? What do you run into?
What additional support would you need to make NGA implementation feasible?
How relevant, necessary and complete did you find the training?

____

____

Item S2: Post-implementation survey amongst health care professionals
Translated from Dutch.

Q16

	Disagree (1)	Somewhat disagree (2)	Neither agree nor disagree (3)	Somewhat agree (4)	Agree (5)	Not relevant to my role
15. I am aware of the reports about the effects of NGA.	○	○	○	○	○	☐
16. The staff agree that the NGA is worthwile.	○	○	○	○	○	☐
17. I value the effects that the NGA has had on my work.	○	○	○	○	○	☐
18. Feedback about the NGA can be used to improve it in the future.	○	○	○	○	○	☐

Q17 Could you briefly elaborate your answers? (optional)

If colleagues do not agree with the NGA, where is the discussion?
If NGA is worth it, what are the most important points of added value?
What adjustments to the NGA would you like?

____

____

End of Block B: integration in routine care (NoMAD questionnaire)

Item S2: Post-implementation survey amongst health care professionals
Translated from Dutch.

Start of Block C: *contribution of NGA to routine care*

Q18 The NGA supports in..:

1. ..the identification and objectivation of, otherwise unnoticed, impairments
[if not applicable for your role: plaese leave score blank]

Strongly disagree — Strongly agree

(0) (1) (2) (3) (4) (5) (6) (7) (8) (9) (10)

2. .. adjusting or supplementing treatment strategies.

Strongly disagree — Strongly agree

(0) (1) (2) (3) (4) (5) (6) (7) (8) (9) (10)

3. ..inform in decision-making for choice of therapy.

Strongly disagree — Strongly agree

(0) (1) (2) (3) (4) (5) (6) (7) (8) (9) (10)

End of Block C: *contribution of NGA to routine care*

Item S2: Post-implementation survey amongst health care professionals
Translated from Dutch.

Start of Block D: instruments of the NGA

Q19 Starting from the 3 just mentioned objectives of the NGA (identifying and objectifying impairments, determining supportive treatment strategies, informing decision-making for choice of kidney replacement therapy).

How relevant are the specific instruments from the test set for your role in kidney patient care?

(Please fill in your judgment and motivation)

	Not relevant at all	Not relevant	Neutral	Relevant	Very relevant	motivation:	Not relevant to my role:
Handgrip strenght	O	O	O	O	O	....	☐
Fall risk assessment	O	O	O	O	O	....	☐
MoCA (cognition)	O	O	O	O	O	....	☐
6-CIT (6-item cognitive impairment test)	O	O	O	O	O	....	☐
Letter Digit Subsitution Test (cognition)	O	O	O	O	O	....	☐
Depression-questions (GDS2/15)	O	O	O	O	O	....	☐
Surprise question *("I would be surprised if patient died after 12 months")*	O	O	O	O	O	....	☐
Frailty score	O	O	O	O	O	....	☐
Comorbidity index (Charlson)	O	O	O	O	O	....	☐
Polypharmacy (>5 medications)	O	O	O	O	O	....	☐
Nutritional status (PG-SGA)	O	O	O	O	O	....	☐
Caregiver burden (Ediz-plus)	O	O	O	O	O	....	☐

Item S2: Post-implementation survey amongst health care professionals
Translated from Dutch.

Q20 Starting from the 3 just mentioned objectives of the NGA (identifying and objectifying impairments, determining supportive treatment strategies, informing decision-making for choice of kidney replacement therapy).

How relevant are the specific tools of the patient-questionnaire for your role in kidney patient care?

(Please fill in your judgment and motivation)

	Not relevant at all	Not relevant	Neutral	Relevant	Very relevant	motivation:	Not relevant to my role:
Katz ADL-6 (functioning)	O	O	O	O	O	....	☐
Lawton iADL (functioning)	O	O	O	O	O	....	☐
Optimism check	O	O	O	O	O	....	☐
Quality of Life (SF-12)	O	O	O	O	O	....	☐
Dialyse symptom index	O	O	O	O	O	....	☐

End of Block D: instruments of the NGA

Item S2: Post-implementation survey amongst health care professionals
Translated from Dutch.

Start of Block E: Reasons for successful or limited implementation

Q21 The reasons below may have been reason for good or limited implementation. Please indicate, from your experience, were these reasons present when the NGA was performed in your hospital?

Patient-related factors

	Not at all present — Strongly present (1 2 3 4 5 6 7 8 9 10)	Not applicable to my role
The purpose and results of the tests were discussed in detail with the patient		☐
Patients were willing and available for the geriatric assessment		☐
The NGA is too much of a burden for many patients		☐
NGA was performed to a limited extent because many patients had low health literacy or due to a language barrier		☐

Multidisciplinary cooperation

	Not at all present — Strongly present (1 2 3 4 5 6 7 8 9 10)	Not applicable to my role
Good cooperation with geriatrics department		☐
Reluctance in the Nephrology department to involve geriatrics/elderly care in routine care		☐
Multidisciplinary consultation and reports in which NGA outcomes and treatment policy were discussed		☐
Support from other disciplines (e.g. dietitian, social worker) in the administration and interpretation of NGA		☐
Loss of geriatric knowledge and practical skills (for example due to team changes)		☐

Item S2: Post-implementation survey amongst health care professionals
Translated from Dutch.

Organizational factors

	Not at all present — Strongly present (1 2 3 4 5 6 7 8 9 10)	Not applicable to my role
Suitable (and trained) personnel were sufficiently available to administer the NGA		☐
The outpatient schedule was easy to adjust for NGA administration		☐
Management supports the implementation of the NGA		☐
Time constraints restricted carrying out the NGA		☐
Lack of budget is a reason to carry out NGA less often or adequately.		☐
The sum-score forms in the dashboard (available from July 2020) were helpful		☐

Q22 Do you have any additional explanation about reasons for successful or limited implementation? (optional)

____

____

____

Item S2: Post-implementation survey amongst health care professionals
Translated from Dutch.

Q23 What are the 3 most important for limited and successful implementation of NGA in your hospital?

Choose from the above reasons or fill in yourself. Please drag the reasons to the boxes.

Discuss purpose and goal with patient
Patient burden
Low health literacy or language barrier
Patient willingness to participate
Cooperation with geriatrics department
Multidisciplinary meetings and repots
Execution of NGA by nurses or nurse practitioners
Execution of NGA other disciplines: e.g social work, dietician, ...
Loss of knowledge (e.g. due to team changes)
Outpatient schedule could (not) be adjusted
Involved key person, i.e. discipline…
Management support
Time (constraints)
Budget (constraints)
(non) availability of digital forms with NGA scores
Other reasons:....

3 reasons for limited implementation:
1.
2.
3.

3 reasons for successful implementation
1.
2.
3.

End of Block: Deel E: Redenen voor goede of minder goede implementatie van NGA in routine zorg

Item S2: Post-implementation survey amongst health care professionals
Translated from Dutch.

Start of Block F: verbeteringen van het geriatrisch assessment

Q24 What is your opinion on the frequency and target group of the geriatric assessment in the POLDER study?

	too little/narrow	just right	too expanded
Frequency: once a year	○	○	○
Target group: age limit 70+	○	○	○
Target group: kidney function <20mL/min/1.73m2	○	○	○

Q25 Please elaborate briefly (optional):

____

____

____

Q26 Finally, do you have any other suggestions or comments about the nephro-geriatric assessment or the POLDER study?

____

____

____

This is the end of the questionnaire. If you have any questions or comments, please refer these to <email address>.

Thank you very much for your cooperation!

End of Questionnaire:

Table S1: Constructs of integration in work routines, assessed with the NoMAD tool.

Construct	Explanation	Contains subconstructs (question number)[a]	Question label in survey [b]
Sense making	how people make sense of what needs to be done.	*Differentiation (*SM1), *Communal specification* (SM2), *Individual specification* (SM3), *Internalization* (SM4)	Q10
Cognitive participation	how relationships with others influence outcomes	*Initiation* (CP1), *Legitimation* (CP2), *Enrolment* (CP3), *Activation* (CP4)	Q12
Collective action	how people work together to make practices work	*Interactional workability* (CA1), *Relational integration* (CA2/3), *Skill set workability* (CA4), *Contextual int*egration (CA5/6)	Q14
Reflexive monitoring	how people assess the impact of NGA	*Systemization* (RM1), *Communal appraisal* (RM2), *Individual appraisal* (RM3), *Reconfiguration* (RM4)	Q16

[a] Refers to the questions as shown in Figure 2.

[b] Refers to the questions as shown in Supplemental file 2.

Table S2: Implementation outcomes per center

Center number	Patient inclusion	Intervention components			
		Execution of provider-administered NGA instruments		Multidisciplinary team meeting	
	(n)	Conducted by disciplines	combined with regular hospital visit *or* separate visit	NGA outcomes discussed	Disciplines involved
1	10	RN	combined	At indication [a]	N, NP, NN, G, SW, D
2	22	GN, D	combined	Yes	N, NN, G, GN, SW, D [b]
3	21	NP/NN, D	combined	Yes	N, NP, NN, G, SW, D
4	30	NP	combined & separate [c]	Yes	N, NP, NN, SW, D
5	19	NN	separate /combined [d]	Yes	N, NN, G
6	10	G	combined & separate [c]	Yes	N, NP, NN, G [b], SW, D,
7	26	NP, G	combined	Yes	N, NP, NN, G, SW, D
8	15	NP	combined	Yes	N, NP, G, SW
9	13	GN/G [e]	separate	Yes	N, NP, NN, G, P, SW
10	25	NN/GN [f]	combined	No/Yes [g]	N, NP, NN, SW, D
Total	191				

Abbreviations: BL: baseline; D: dietician; G: geriatrician (or internist-geriatrician); GN: geriatric nurse; N: nephrologist; NN: nephrology nurse; NP: nurse practitioner (nephrology); P: psychiatrist; PQ: patient-questionnaires; RN: research nurse; SW: social worker.

[a] Only for patients in whom there were doubts on physical and cognitive functioning.

[b] Discipline not always present at multidisciplinary team meeting.

[c] Either combined or in a separate visit, often in combination with educational- and/or doctors' consultation.

[d] Initially separate visits but later these were combined.

[e] Initially conducted by geriatric nurse, later by internist-geriatrician, in future disposition to be done by a nephrology nurse (practitioner).

[f] Geriatric practices were conducted by a geriatric nurse who took over from the nephrology nurse.

[g] During/subsequent to the study period a multidisciplinary team meeting was developed.

Table S3: Completeness of NGA instruments.

NGA instruments			Patient questionnaire n=187		Provided-administered test-set n=190	
Domain	Instrument	Type	Filled in by n (%)	Completed by n (%)	Assessed for n (%)	Completed for n (%)
Functional status	Activities of daily living (Katz ADL-6)	P	186 (99)	183 (98) [d]		
	Instrumental activities of daily living (Lawton)	P	187 (100)	172 (92) [d]		
	Handgrip strength	T			180 (95)	164 (86) [c]
	Fall risk assessment	T			177 (93)	173 (91)
Cognitive functioning	Montreal Cognitive Assessment	T			189 (99)	189 (99)
	6-item Cognitive Impairment Test	T			184 (97)	184 (97)
	Letter Digit Substitution Test	T			185 (97)	185 (97)
Psychological status/mood	Whooley-questions / Geriatric Depression Scale-15	T			190 (100)	190 (100)
	Life Orientation Test-Revised	P	185 (99)	160 (86) [d]		
PROM's	HR-QoL: 12-item Short Form Health Survey	P	187 (100)	153 (82) [d]		
	Dialysis Symptom Index	P	187 (100)	135 (72)		
Somatic status	Surprise question	T			181 (95)	139 (73) [c]
	Clinical Frailty Score, [a]	T			190 (100)	NA
	Charlson Comorbidity Index [a]	T			190 (100)	NA
	Polypharmacy (≥5 medications) [a]	T			190 (100)	NA
Nutrition	Patient-Generated Subjective Global Assessment [b]	P/T	186 (99)	76 (41)	156 (82)	125 (66) [d]
Social	Caregiver burden: EDIZ-plus	CG	121 (64)	113 (60) [d]		

Abbreviations: P, patient questionnaire; T, provider-administered test-set; HR-QoL, health-related quality of life

[a] Clinical Frailty Score, Charlson Comorbidity Index, Polypharmacy were instruments derived from electronic patient file.

[b] PG-SGA consists of a part filled in by the patient and a part filled in by the health-care provider.

[c] Handgrip strength: less than 3 measurements done. Surprise question: filled in by less than 3 different disciplines (nephrologist, geriatrician, nurse practitioner, dialysis nurse)

[d] Incomplete instrument restricted use of the instrument (e.g. because no total score could be generated).

Table S4: Determinants of implementation identified in qualitative analysis

Determinant / NPT (sub) construct	Summary of qualitative content (Figure S1)	Quote	Supporting evidence [a]
**Sense making**			
Differentiation	NGA differs from usual care in providing objective insights, identification of unnoticed impairments. This leads to better conversations on suitable care (support) between HCW and patients, and among HCWs.	Q1<br>Q2	*NGA gives us the opportunity to get a more complete picture of a patient and not just medical problems. In addition we discuss life goals and the impact of treatment choices on these goals.* (nurse practitioner)<br>*Being aware of a patient's functionality in all domains is a valuable tool which can enhance conversations about future KRT decisions. Patients appreciate that we assess them in this [NGA] manner and can more readily accept advice based on results.* (nephrologist)
Communal specification	In some centers the goal of NGA was widely acknowledged, but not everywhere.	Q3<br>Q4	*Due to the POLDER study we recognize the importance [of NGA] for this older patient group and that is why we have set up a [nephrology]geriatric committee with a geriatrician and psychiatrist.* (nurse practitioner)<br>*NGA is carried out for patients with kidney failure, not yet started on dialysis. I myself am not involved further and it doesn't impact my profession. Neither are my dialysis colleagues involved in the screening. Most of them don't even know about NGA.* (nephrology nurse)
Individual specification	NGA is perceived informative for patients' decision making on KRT e.g., to dispel doubts. Although a few respondents have doubts on the relevance of NGA and its value for the decision-making process for all patients.	Q5<br>Q6<br>Q7	*It [NGA] helps us to get a better picture of a patients activities of daily living. Sometimes it helps when making KRT decisions or to dispel doubts.* (nurse practitioner)<br>*Due to NGA we have a more objective picture [of a patient], previously our point of departure was instinct our "gut feeling". As a social worker I can give support to issues which are relevant within my discipline such as burdened caregiver, social isolation. Importantly, the decision making process can be impacted by NGA outcomes (social worker)*<br>*In critical cases it [NGA] plays a role in decision making about whether to start or not to start KRT. We don't really know as yet how the treatment is influenced, what is reversible and what is not.* (nephrologist)
Internalization	NGA is of value for consideration of treatment options, to emphasize quality of life and provide suitable care. A minority of the respondents do not think NGA is of value; as it would not lead to concrete advices and only sporadically contributes to the decision-making process.	Q8<br>Q9	*The frailty which you are in practice often aware of is substantiated with NGA results in all domains. Sometimes even unexpectedly. We can take this [results NGA] into consideration and offer support where needed. Thus the quality of life of each individual patient comes first and foremost and is the point of departure [for treatment choice]* (nephrologist)<br>*There was no clear feedback and particularly no specific advice (other than numeration of NGA results). Sporadically, it [NGA results] contributed to decision making, whereby before evaluation the decision had already been made.* (nephrologist)
**Cognitive participation**			
Initiation	Key persons are those who execute the NGA and maintain the connection between nephrology and geriatrics; i.e. nephrology nurse (practitioner) with geriatric affinity, sometimes a nephrologist, or a geriatric nurse or -specialist. It is of risk that this often relies on one person.	Q10<br>Q11<br>Q12	*A nurse practitioner or physician assistant who has affinity with older patients plays a crucial role in building a bridge between the nephrologist and geriatrician.* (geriatrician)<br>*Key figures are the ones who perform the assessment. They are responsible for feedback to the multidisciplinary team.* (social worker)<br>*I was given enough time to perform NGA (particularly for the POLDER study). However I am alone and if we want to implement this [NGA] into daily practice then more colleagues are needed. The demand to carry out NGA is increasing.* (nephrology nurse)
Legitimation	While one respondent described that NGA is not for all CKD patients considered legitimate; another stated that the increasing number of frail older CKD patients legitimates standardized NGA.	Q13<br>Q14	*I do see the value [of performing NGA] for a specific group of patients (not based on age but on estimation of frailty), however I miss the follow-up and specific advice.* (nephrologist)<br>*I see so many frail older patients who have been referred and I do think it is useful for both the practitioner and the patient to invest time in deciding together what is the best treatment.* (nephrologist)

Determinant / NPT (sub) construct	Summary of qualitative content (Figure S1)	Quote	Supporting evidence [a]
Enrolment	Working together with the geriatrics nurses or specialists is acknowledged.	Q15	*In my hospital the goal is to employ a trained geriatric nurse on every ward so that quality of care for older patients can be improved, certainly now as the number of older patients is steadily increasing.* (nurse)
Activation	Support for the intervention is hampered by doubts about its usefulness in all patients.	Q16	*I really have difficulty with implementation of a standardized NGA for ALL patients above a certain age, and I believe that HCW who carry out NGA should be very careful about making statements about whether or not to start dialysis.* (nephrologist)
		Q17	*I support the NGA and perform it too because firstly, it offers the patient and their family better support and coaching. Secondly, it gives a lot of information about the expected treatment, degree of frailty and expected care level and prognosis.* (nurse practitioner)
		Q18	*In the meantime, more frequently geriatric assessment is requested for patients from the out-patients kidney failure clinic. We would like to see that it [NGA] becomes part of routine care. The nephrologists support the NGA and requests are becoming more frequent.* (nurse)
**Collective action**			
Interactional workability	Although some stated that NGA fits well in existing outpatient care (through either standard geriatrics visit or integrated in nephrology visit with reference to geriatrics if needed), some centers needed to ask a research nurse and it was noted that not all patients were screened.	Q19	*The NGA is integrated into the outpatient clinic workflow and care pathway.* (nurse practitioner)
		Q20	*Over the past few years NGA has become more integrated into our workflow, including patients who do not participate in a study. Referral on to the geriatrician after NGA occurs regularly. The conversation with the patient and their family about KRT choice is more validated.* (nurse practitioner)
Relational integration	Confusion on focal point or which specialism is in lead may disturb working relationships. Multidisciplinary collaboration, involvement and communication is needed, and is sometimes subject for improvement. Multidisciplinary team meetings were acknowledged.	Q21	*To a degree, it disrupts work relationships because other specialists (geriatrician) discusses nephrology related treatments with the patient. I don't know precisely how these discussions go, particularly if a patient has questions [about KRT], are they then referred to the nephrologist with their questions?* (nephrologist)
		Q22	The discussion [amongst HCW] is due to the overlap [of tasks] with other disciplines, e.g. social worker: what is the difference between NGA and PROMS or a well-being measure? Can another discipline carry out NGA, e.g. a nurse. Who decides/interprets when a geriatrician is necessary? (nurse practitioner)
Skill set workability	Lack of knowledge and experience with NGA may hamper communal appraisal. Some said that inter-hospital knowledge exchange and (continued) training is needed, e.g. for low illiterate patients.	Q23	*Not all our colleagues have experience using NGA and because of this they don't see the advantages of the assessment. There is a lack of knowledge.* (nephrology nurse)
		Q24	*The initial training was certainly relevant, the intention was to repeat the training and the proficiency test because of the risk of faulty routine setting in. But also to carry out work visits to exchange experience with other hospitals. (nurse practitioner)*
Contextual integration	Management mostly supports NGA but not in every center. Lack of resources (i.e. time, availability of (dialysis) nurses, and budget for multidisciplinary cooperation) was noticed frequently. Information dissemination could be optimized, e.g. by software.	Q25	*The dialysis center management team does recognize the importance of good elderly care and facilitates extra education, work-shops and clinical education.* (nurse)
		Q26	*The key figures on the nephrology plus outpatients clinic are the nurses, unfortunately they are given too little time to perform their work properly and this is a recurrent issue which is discussed at meetings with the unit coordinator.* (geriatric nurse)
		Q27	*The most important missing resources are time and manpower, and shortage of dialysis nurses is the most pressing because they are needed on the kidney failure outpatients clinic.* (geriatrician)
		Q28	*During the POLDER study it became clearer what the benefits of the questionnaires, the tests, the results and extra information meant. This was all discussed at the multidisciplinary meetings. And we experienced an increasing awareness of the added value of NGA and the improvements due to consultation with the geriatrician.* (nurse practitioner)
		Q29	*There was however long term leave of absence of one HCW and that left us with only one other person who could do NGA which made things more complicated.* (nurse practitioner)
**Reflexive monitoring**			

Determinant / NPT (sub) construct	Summary of qualitative content (Figure S1)	Quote	Supporting evidence [a]
Systemization	Some noted that effectiveness of NGA has not been established. According to others effectiveness is sufficiently proven in medical fields (e.g. oncology) or from own experience.	Q30	*I`m not aware of any studies which compare the value of NGA versus the judgement/appraisal of an experienced nephrologist whether dialysis is beneficial for the individual patient.* (nephrologist)
		Q31	*The importance of performing CGA or NGA is no longer a under discussion at the geriatric out-patient clinic because we already have proof of its value, also its value in oncology*. (geriatric nurse)
Communal appraisal	Some noticed more demand for NGA, and improvement of multidisciplinary cooperation due to NGA. But the value is not seen by everyone; mostly due to needed time-investments, burden for patients, or lack of concrete outcomes.	Q32	*Colleagues do not always regcognise the value [of NGA] and only see the disadvantages: the extra time it costs for patients and family.* (nephrologist)
		Q33	*Colleagues are searching for more tangible outcomes and the effect on treatment choice [of NGA results] as it's not a straightforward choice, only in extreme outcomes.* (nephrologist)
		Q34	*Colleagues from geriatrics do see the added value [of NGA] because they have experienced it in other combinations e.g. orthopedic-geriatric care; some nephrology colleagues see the added value and some recognize the limitations and time investment.* (geriatrician)
Individual appraisal	Most respondents value the effects of NGA; i.e. holistic view on frailty, improved patient-conversations and personalized care. But some noted to see no clear effect of NGA.	Q35	*There is no clear feedback and especially indisputable advice other than a summing-up of results. In practice this [NGA] contributes sporadically to decision making, whereby this information [decision about KRT] was already known prior to evaluation* (nephrologist)
		Q36	*I find it an important part of patient assessment and for individualized tailored care to support treatment choice. However it isn't as yet being performed with every patient who is eligible.* (nephrology nurse)
		Q37	*The added value is we have more insight than merely medical facts.* (nephrologist)
Reconfiguration	Some parts of NGA may not fit all patients, need for adjustments for illiterate non-Dutch speaking persons. Further development of NGA is needed said some; e.g. through multidisciplinary team meetings, availability of geriatrics, potential use of shorter screening tool for full NGA.	Q38	*The extensiveness of the assessment and the applicability in daily practice needs adjusting. Some of the assessment domains are not suitable for everyone*. (geriatrician)
		Q39	*I have been given tools to start a patient conversation. Let's hope that a DIALOGICA [study] results in an abridged version of NGA.* (nephrologist)
		Q40	*Perhaps adjustments to the test set is possible, depending on the patient and the hospital setting, e.g. targeting other languages, level of health literacy, or use of The 6-CIT versus The MoCA.* (nurse practitioner)
**Other determinants**			
Patient burden		Q41	*Quite often patients found the questions too difficult. Some questions were found to be too long and these comprised different parts*. (nurse practitioner)
		Q42	*A considerable number of patients experience hospital visits as very burdensome and therefore decline to participate in a study. (geriatrician)*
		Q43	*I think that we can break the taboo of assessment and implement it more broadly, by giving it a different name or taking it out of the "geriatric" realm, and then patients will be more inclined to participate.* (nurse practitioner)
		Q44	*By less linking of it [NGA] to age you already take away the taboo, they don't want to be categorized as "geriatric patient" and are sometimes afraid of being tested for cognition.* (nephrologist)

[a] All selected illustrative quotes were translated from Dutch to English by a native speaker (NB) using back-translation.

Abbreviations: 6-CIT, 6-item cognitive impairment test; HCW, health care worker; KRT, kidney replacement therapy; MoCA, Montreal cognitive assessment; NGA, nephrology-tailored geriatric assessment; PROMS, patient-reported outcome measure.

Table S5: Outcomes of qualitative analysis on the relevance of each NGA instrument.

Domein	Instrument	Summary	Supporting quotes (respondents' discipline: Likert-scale rating)
Physical	KATZ-ADL & Lawton iADL	Identification of frailty and assessment of care needs	*Basic functioning is important in frailty assessment.* (geriatrician: very relevant)<br>*This [physical functioning] domain could be targeted with supportive interventions.* (geriatrician: very relevant)<br>*I seldom use this in my daily practice.* (nephrologist: neutral)
	Handgrip strength	Measuring deterioration of physical status, predictive value for interventions	*Indicates ability to withstand invasive procedures.* (nurse practitioner: relevant)<br>*I don't use it at all.* (nephrologist: not relevant)
	Fall risk	Provides insights in physical functioning and need for preventive interventions	*Because of sarcopenia diagnostics. (dietician: very relevant)*<br>*Each dialysis patient has a higher fall risk. (nurse practitioner: neutral)*
Cognitive	MoCA	Identification of unrecognized impairments, helpful in decision-making and patient care	*Provides insight into minor impairments of executive functions.* (nurse practitioner: very relevant)<br>*Unnoticed cognitive impairments become apparent.* (nurse practitioner: very relevant)<br>*It's not something I have experience with, in terms of interpretation and use.* (nephrologist: very relevant)
	6CIT	Provides information about cognition. Less sensitive and no added value compared to MoCA	*Sensitivity is poor/insufficient.* (geriatrician: not relevant)<br>*It is only aimed at screening and that is insufficient in this patient group.* (nurse practitioner: neutral)<br>*Short test, not always the same outcome in my view as the MoCA.* (nurse practitioner: very relevant).
	LDST	Limited use in practice	*Subtle indication of problems, e.g. choice for peritoneal dialysis.* (nurse practitioner: relevant)<br>*Difficult, especially with language problems.* (nurse practitioner: neutral)<br>*It is more research oriented.* (geriatrician: neutral)
Psychosocial	GDS	Initiating conversations and relevant for treatment choice, provides insights in need for interventions	*Being able to start a conversation about mood is important, especially when difficult choices have to be made.* (nurse practitioner: very relevant)<br>*Useful, but sometimes language problem.* (nurse practitioner: very relevant)
	Optimism (LOT-R)	Insight into resilience and motivation. Little used in practice.	*Indicative of a patient's attitude to life is alive and whether he will be able to deal with a burdensome treatment.* (nurse practitioner: relevant)<br>*I wonder about the added value compared to the Geriatric Depression Scale.* (nephrologist: not relevant)<br>*Not often used in practice.* (geriatrician: neutral)
PROM's	Health-related quality of life (SF-12)	Relevant for discussion of treatment choice	*I use this too little in practice.* (nephrologist: neutral)<br>*Especially important to start a conversation about whether or not to dialyze.* (geriatrician: relevant)
	Dialysis Symptom Index	Very useful to decide on supportive interventions and treatment choice.	*Directly applicable in outpatient clinics, focusing on specific complaints.* (nephrologist: very relevant)<br>*Does often provides new insights.* (geriatrician: very relevant)
Somatic	Surprise question	Awareness with regard to prognosis for the healthcare provider, but the predictive value is difficult to interpret.	*Moderate predictive value.* (nephrologist: neutral)<br>*This still feels most familiar: to listen to your 'gut feeling*' (nephrologist: very relevant)
	Clinical Frailty Scale	Relevant to decision on treatment choice and supportive interventions	*By regularly scoring this, you can see the follow-up over time and therefore notice when the patient is deteriorating.* (nurse practitioner: very relevant)<br>*Makes this more transparent and helps to speak the same language.* (nephrologists: relevant)
	Charlson Comorbidity Index	Little used or already part of standard care. Important for choice of therapy.	*I don't need to have a score if I know the patients' history.* (nephrologist: not relevant)<br>*Is scored anyway.* (nephrologist: very relevant)<br>*Plays a part in the treatment choice for renal failure.* (nurse practitioner: relevant)
